# Distribution Prediction and Adaptability Analysis of Section *Camellia* Plants (*Camellia* Genus) in China Based on the MaxEnt Model

**DOI:** 10.1002/ece3.71365

**Published:** 2025-04-26

**Authors:** Weihao Gu, Xu Xiao, Zhaohui Ran, Chao Yan, Dongzhen Jiang, Lei Zhou, Mingtai An, Zhi Li

**Affiliations:** ^1^ College of Forestry Guizhou University Guiyang China

**Keywords:** climate change, MaxEnt model, potentially suitable area, sect. *Camellia*

## Abstract

Sect. *Camellia* plants, widely distributed across southern China, hold significant economic value through their dual applications in landscape greening, ornamental horticulture, and oilseed production. However, with rapid changes in the global climate, it is becoming increasingly important to study the habitat distributions of species and the factors influencing their adaptations. Using the maximum entropy model, we predicted the past, present, and future distribution areas of suitable habitats for sect. *Camellia* under different climate scenarios. The results revealed that under current climate conditions, the total suitable area of sect. *Camellia* was 17.04 × 10^5^ km^2^, and the highly suitable area was 1.95 × 10^5^ km^2^. The distribution of sect. *Camellia* was strongly influenced by key environmental factors, such as the maximum temperature in the hottest month (Bio5), the minimum temperature in the coldest month (Bio6), the annual difference in temperature (Bio7), and the slope (Slope). In view of future climate change, the suitable distribution center of sect. *Camellia* is expected to shift to higher latitudes and may undergo northward movement to adapt to new environmental conditions, leading to an expansion of the total suitable area.

## Graphical abstract

Sect. *Camellia* is mainly distributed in the southern regions of China; this research underscores its dual role in esthetic enhancement and industrial oil provision. Using the maximum entropy model, our study unravels the historical, contemporary, and projected spatial landscapes of sect. *Camellia* under diverse climate scenarios, pinpointing a substantial 17.04 × 10^5^ km^2^ suitable zone with a concentrated 1.95 × 10^5^ km^2^ core area, significantly swayed by Bio5, Bio6, Bio7, and Slope. Anticipating future climate shifts, the distribution of sect. *Camellia* nucleus foresees northerly migration toward higher latitudes, expanding its ecological footprint—a revelation vital for conservation strategies amid global warming.

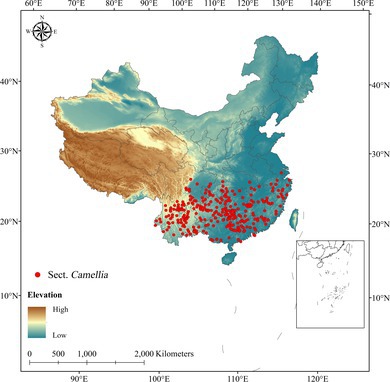

## 1 Introduction

Climate change has always been an important driver of species evolution and distributional shifts in global development (Li, Shao, et al. 2022). Today, warming temperatures, uneven precipitation distribution, increased frequency of extreme weather events, and changes in precipitation patterns, especially increases in drought events, have threatened the ability of plants to access water, affecting their growth and reproduction, and have also put great pressure on plant species that depend on specific environmental conditions for survival (Deng 2020; Wu and Wiens 2022; Plos et al. 2024). However, environmental changes triggered by global warming have significantly affected the growth cycle, flowering time, fruit ripening time, and seed dispersal efficiency of plants, which in turn have affected the stability of the entire ecosystem (Niu et al. 2024). More seriously, some plant species are facing an existential crisis and sometimes even the risk of extinction due to their inability to rapidly adapt to drastic environmental changes (Zhao et al. 2024). Therefore, exploring the response mechanisms of plants to climate change and predicting their future distribution trends are crucial for developing effective biodiversity conservation strategies (Liang 2020). To address this challenge, researchers have developed a variety of ecological niche modeling methods in recent years. Ecological modeling for predicting species distributions includes Ecological Niche Factor Analysis, Bioclimatic Analysis System, Genetic Algorithm for Rule Set Production, and maximum entropy (MaxEnt) model (Wang, Xie, et al. 2023). Among them, MaxEnt has higher modeling accuracy and has been widely used. The MaxEnt model was introduced by Phillips et al. (2006), laid the theoretical groundwork for subsequent ecological studies, and it has been instrumental in analyzing species distribution and habitat suitability (Phillips and Dudík 2008). The MaxEnt model is based on the principle of MaxEnt in statistical physics, and the model estimates the potential distribution probability of a species under a given environmental variable by minimizing hypothesis bias (Cai et al. 2024; Li, Qin, et al. 2024; Yang et al. 2024). Compared with traditional interpolation methods, MaxEnt is able to address nonlinear relationships, capture complex spatial heterogeneity features, and generate reliable predictions even with limited species record data. In addition, the model allows for the incorporation of multiple environmental variables, including but not limited to topography, climate indicators, and soil type, thereby increasing the comprehensiveness and accuracy of the prediction results (Li, Cao, et al. 2024). When combined with GIS technology, the MaxEnt model can visualize the geographical pattern of species distributions, providing strong data support for ecological management and decision support (West et al. 2016; Jia et al. 2024).

Sect. *Camellia* belongs to the genus *Camellia* and is an important member of the *Camellia* genus with high ornamental and economic value. This group comprises 57 Asian species and 55 in China (Zhang and Ren 1998), with *Camellia* concentrated south of the Yangtze River (Tianlu and Wenju 1996) in high‐altitude mountains of Guizhou, Sichuan, Zhejiang, Guangdong, and Hunan (Tian 2001; Xie 2010; Zhang 2007). In the genus *Camellia*, the species included in the IUCN Red List are: Critically Endangered (CR) *Camellia impressinervis* and *Camellia azalea*; Endangered (EN) *Camellia indochinensis* var. *tunghinensis* and *Camellia amplexicaulis*; Vulnerable (VU) *Camellia euphlebia*; and Near Threatened (NT) *Camellia flavida* var. *patens*. Meanwhile, it is valued for its ornamental blooms, premium tea leaves, and ecological significance, thriving at 500–2000 m elevation in acidic soils (pH 4.5–6.5), co‐occurring with evergreen broadleaved species like *Camphora officinarum* and *Phoebe zhennan*. In addition, they grow together with some shrubs and herbs, such as *Rhododendron* simsii, ferns (Shi 2003; Tang et al. 2022).

Meanwhile, plants in sect. *Camellia* contain a variety of bioactive components, such as flavonoids, triterpenoids, and organic acids (Wang et al. 2012). The leaves and flowers are rich in nutrients like tea polyphenols, amino acids, and vitamins, possessing multiple medicinal values in terms of antioxidant, anti‐inflammatory, anticancer, and antibacterial effects (Shen et al. 2022). In addition, the unsaturated fatty oil contained in its seeds, commonly known as “*camellia* oil,” is particularly suitable for patients with cardiovascular and cerebrovascular diseases to consume, demonstrating the potential of sect. *Camellia* in terms of edible value (Gao et al. 2024). Current research on this taxon is focused mainly on taxonomy. In the mountainous areas of southern China, sect. *Camellia* not only beautifies the natural landscape but also promotes local economic development and cultural heritage (Li and Zheng 2008). Deng et al. (2006) confirmed Chang Hongda's classification of sect. *Camellia*, showing that morphological traits closely correlate with DNA sequence similarities, thus providing molecular evidence for the taxonomy. The research on sect. *Camellia* also includes genetic and phylogeny studies. Tian et al. (2008) studied the phylogenetic relationships of the Internal Transcribed Spacer sequences of plants in sect. *Camellia* using ITS sequences, integrating traditional morphological results, comprehensively analyzing and studying their systematic classification, exploring and assessing the taxonomic position, attribution, and evolutionary history of some species, and reconstructing the ITS phylogenetic tree to provide a new molecular basis. Ni et al. (2007) analyzed the affinities of sect. *Camellia* by microscopically examining their pollen. Currently, most of the microstudies of sect. *Camellia* are taxonomic studies, such as Shen et al. (2008) using infrared spectroscopy, which provided valuable information for origin and evolution studies. However, the impact of global climate change, especially alterations in temperature and precipitation patterns, may disrupt the survival balance of this species and threaten its genetic diversity and ecological roles (Wang et al. 2024).

In this study, we aim to predict the distribution and analyze the adaptability of sect. *Camellia* plants in China (55 species) using the MaxEnt model. We combined geographic distribution data and environmental variable data to identify the current optimal adaptation regions and simulate potential distributional changes under future climate change scenarios. Our objectives are to: (1) utilize the MaxEnt model to predict the distribution of sect. *Camellia* and assess its adaptability under current environmental conditions, (2) simulate the potential impacts of climate change on the distribution and adaptability of sect. *Camellia*, and (3) evaluate the dominant environmental factors influencing the distribution pattern of sect. *Camellia and* their evolution with climate change.

The outcomes of this study will enhance our understanding of the biology and ecological niche of sect. *Camellia* and provide a scientific foundation for habitat assessment, population migration prediction, and species conservation and exploitation.

## 2 Materials and Methods

### 2.1 Data Collection and Treatment

**FIGURE 1:**
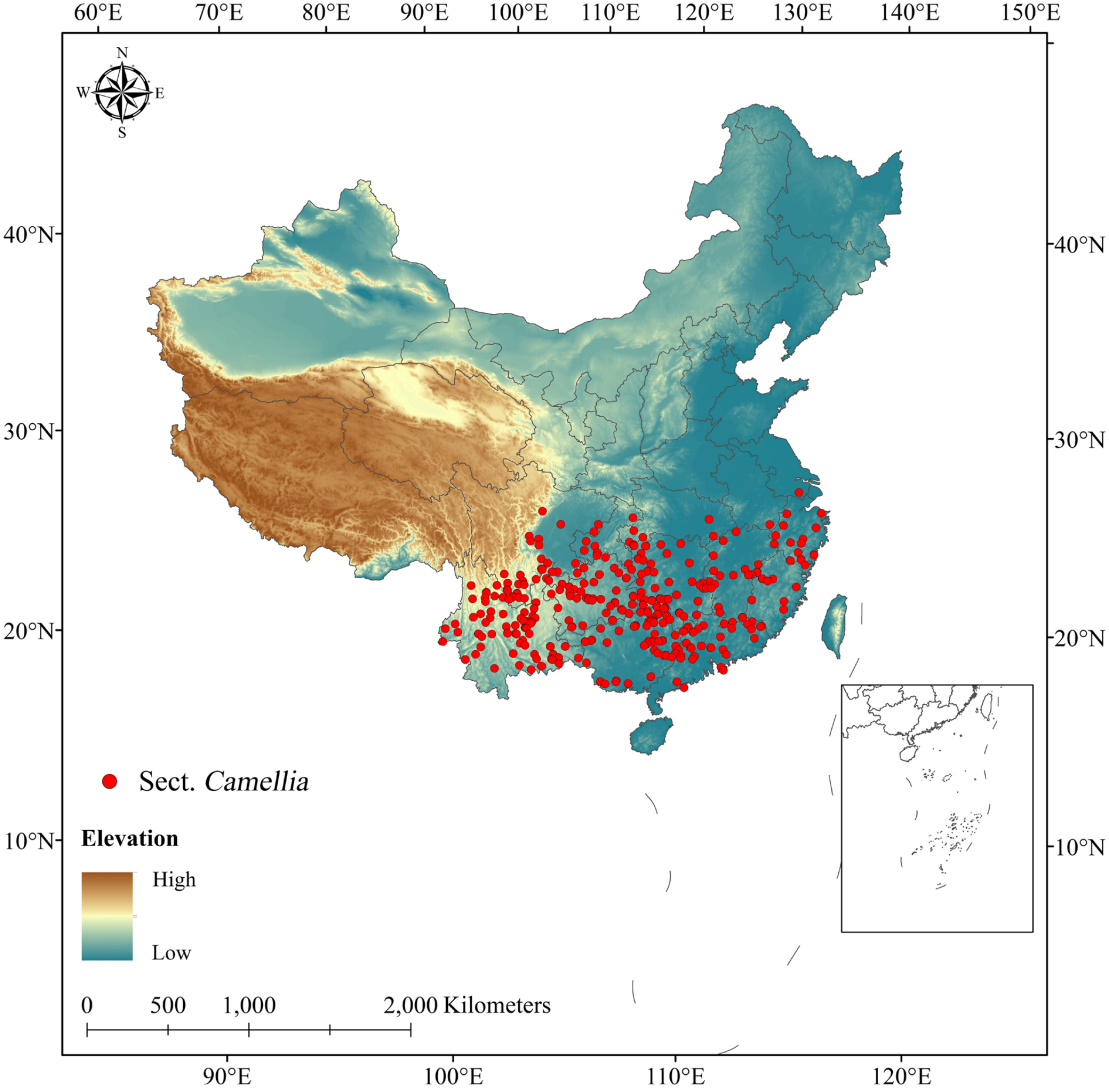
Distribution points of sect. *Camellia*.

The spatial distribution of the plants within sect. *Camellia* is primarily delineated through a comprehensive analysis of plant specimens, which includes not only our own field‐collected samples but also data sourced from several reputable databases. These 55 species' resources in China encompass the Chinese Virtual Herbarium (https://www.cvh.ac.cn/), the Global Biodiversity Information Network (GBIF, https://www.gbif.org/), and the Flora of China (https://www.iplant.cn/foc/). The integration of our field observations with these extensive databases provides a robust foundation for understanding the ecological patterns and biogeographical affinities of *Camellia* species. We were able to acquire a preliminary set of 580 distribution records for the plant species of sect. *Camellia*. The collected distribution data were categorized based on species name, longitude coordinates (labeled X) and latitude coordinates (labeled Y). To address the issue of spatial autocorrelation in species distribution data within localized areas, we applied a rigorous data‐thinning approach using the spThin package in R. This method involved retaining only one distribution record per 10 km ×10 km grid, thereby eliminating duplicates and reducing the influence of spatial proximity on our analysis (Aiello‐Lammens et al. 2015; Wang, Duan, et al. 2023). Finally, we obtained 391 sample distribution points of sect. *Camellia* (Figure 1). In addition, these data were organized and stored in .csv file format for subsequent application in the MaxEnt model (Wen et al. 2022).

### 2.2 Acquisition and Screening of Environmental Data

Environmental variable selection primarily focuses on their impact on species distribution and the spatial correlation among them (Peterson et al. 2011; Zhang, Jiang, et al. 2019). Twenty‐two environmental variables were selected for analysis in this study and cover seven key periods: the last interglacial (LIG), the last glacial maximum (LGM), the middle Holocene (MH), the Modern (Current), and the next three projected phases—between 2040 and 2060 (2050s), 2060 and 2080 (2070s), and 2080 and 2100 (2090s). Our environmental dataset includes 19 bioclimatic variables (BIO1–BIO19) and 3 terrain indicators: Aspect of slope (Aspect), Elevation (Dem) and Slope (Slope) (Table 1). This study compiled 19 climate datasets, encompassing both current (1970–2000) and future (2041–2100) scenarios, sourced from WorldClim (http://www.worldclim). Additionally, topography‐related data were also retrieved from the same repository. Representative concentration pathways (RCPs) under the CCSM4 model, which is more effective in simulating China's climate, were selected for future climate data: RCP2.6, RCP4.5, and RCP8.5. These pathways correspond to low, medium, and high greenhouse gas emission intensities and climate forcing levels, respectively (Zhang et al. 2021). All environmental factors were uniformly processed by ArcGIS with a resolution of 30″ (1 km ×1 km). For the simulation of atmospheric circulation, we chose the second‐generation medium‐resolution climate system model (BCC‐CSM2‐MR_2.5) developed by the National Climate Center of China, which has demonstrated good performance in simulating temperature and precipitation scenarios in China (Sang et al. 2019; Su et al. 2021). In order to prevent overfitting due to multicollinearity between environmental factors, we evaluated the contribution of each environmental factor in the model training process and combined the results of Spearman correlation analyses with the (R 4.1.1) “psych” package and excluded the environmental factors with correlation coefficients > 0.8 with low contributions (Table 1) (Gaißer and Schmid 2010; Chen, Long, et al. 2023). As a result of this screening process, 10 environmental variables that were both statistically significant and ecologically important were ultimately identified for use in constructing our model (Table 2).

**TABLE 1:** The importance of environmental variables on the distribution of sect. *Camellia*.

Variable	Describe	Percent contribution (%)	Permutation importance (%)
Bio1	Annual average temperature	0.4	0.1
Bio2	Daily range of average temperature	1.6	2.8
Bio3	Isothermal property	2.6	6.1
Bio4	Seasonal variation coefficient of temperature	3.4	4.1
Bio5	Maximum temperature in the hottest month	1.4	5.7
Bio6	Min temperature of coldest month	14.2	12.5
Bio7	Annual temperature range	3.4	10.3
Bio8	Average temperature in the wettest quarter	0.5	0.4
Bio9	Average temperature in the driest quarter	0.4	0.1
Bio10	Average temperature in the hottest quarter	0.5	0.1
Bio11	Average temperature in the coldest month	2.2	3.5
Bio12	Annual precipitation	14.5	0
Bio13	Precipitation in the wettest month	0.1	6.8
Bio14	Precipitation in the driest month	20.6	14.6
Bio15	Seasonal variation of precipitation	0.4	1.5
Bio16	Precipitation in the wettest season	0.1	0
Bio17	Precipitation in the driest quarter	0.1	4.9
Bio18	Precipitation in the warmest month	0.3	4
Bio19	Precipitation in the coldest season	0.3	9.6
Aspect	Aspect of slope	10.6	0.4
Dem	Elevation	7.8	4.9
Slope	Slope	14.5	7.5

**TABLE 2:** Importance of each dominant environment variable in MaxEnt model.

Variable	Percent contribution (%)	Permutation importance (%)
Bio6	78.2	22.5
Bio7	13.6	5.4
Slope	3.3	8.6
Bio5	1.1	16.3
Dem	0.9	12.3
Bio18	0.8	17.7
Bio8	0.8	5.7
Aspect	0.7	0.1
Bio14	0.4	8.5
Bio12	0.3	3

### 2.3 Model Establishment and Optimization

ENMeval package (R v4.1.1) was used in this study to optimally tune the MaxEnt model to increase the robustness of the data. We gradually increased the value of the regularization multiplier (RM) from the initial value of 0.5 4, increasing it by 0.5 each time. In this process, we also carefully examined six different feature combinations (FCs); including L (linear); LQ (linear and quadratic hybrid); H (fragmentation); LQH (linear, quadratic, and fragmentation hybrid); LQHP (linear, quadratic, fragmentation, and product type mixing); and LQHPT (LQHP type, and threshold type mixing) (Muscarella et al. 2015). During model construction, we tested the parameter combinations using the Kuenm package, and by evaluating the fit and complexity of the model, we selected the optimal parameter combinations based on the deltaAICe values of the Akaike information criterion. We subsequently evaluated and confirmed the prediction accuracy of our model by calculating the area of the region under the receiver operating characteristic curve (ROC curve) (i.e., area under the curve [AUC] value). According to the established evaluation guidelines, an AUC value below 0.6 indicates that the model fails to perform as expected; a value between 0.6 and 0.7 indicates poor model performance; a range between 0.7 and 0.8 indicates fair model performance; when the AUC value falls within the range of 0.8–0.9, we regarded the model as performing well; an AUC value of more than 0.9 not only means that the model exhibits excellent performance but also indicates that the prediction results are highly reliable (Guo et al. 2024).

### 2.4 Model Evaluation

Following data import, the knife‐cut method was applied with logistic regression configuration. The dataset was partitioned into 75% training and 25% testing subsets through randomized allocation. Model parameters were optimized through selective adjustment of RM and FC values while retaining default settings for other variables (Chen, Zhang, et al. 2023). Final validation employed MaxEnt‐derived AUC metrics from ROC curve analysis. Ultimately, we evaluated the accuracy of the data using the area under the ROC curve (AUC value) output from the MaxEnt model. The AUC value serves as a measure reflecting the confidence level of the prediction results and spans values of 0–1. In this range, the higher the AUC value, the more reliable the prediction (Guo et al. 2024; Lai et al. 2022). In addition, commonly used metrics for model evaluation include Kappa and true skill statistic (TSS) values (Cohen 1960; Roldán‐Nofuentes and Regad 2021). Therefore, we calculated Kappa and TSS values to assess model performance, which range from −1 to 1. Higher values of Kappa and TSS indicate higher accuracy and consistency of predictions (Allouche et al. 2006; Zhang et al. 2024).

### 2.5 Grade Differentiation of Suitable Living Areas

At the end of the simulation experiment, we evaluated the accuracy of the MaxEnt model by measuring the area under the subjected graphs with reference to the grading criteria developed by Ye et al. (2021) for *Phoebe bournei* (Hemsl.) Yen C. Yang. Using the natural partitioning method, we categorized the potential growing areas of sect. *Camellia* with the AUC index (in the range of 0–1) into four classes: class I represented unsuitable area (AUC value 0–0.2), class II represented low suitability area (AUC value 0.2–0.4), class III represented high suitability area (AUC value 0.4–0.6), and class IV represented the most suitable area (AUC value 0.6–1).

### 2.6 Spatial Pattern Changes and Centroid Analysis of Species‐Adapted Areas

In this study, we conducted an in‐depth analysis of the distribution of plants in sect. *Camellia* under various levels of climate adaptation, with a special focus on the changes in distribution areas under different climate projection scenarios compared with the current situation. We quantified the areas of expansion, maintenance, or contraction of these plant populations and their spatial ranges under each scenario. Employing the methodological framework established by Lv et al. (2024), ArcGIS binary processing classified habitat suitability using 0.2 probability thresholds (unsuitable: < 0.2; suitable: ≥ 0.2), generating transition matrices that quantified spatiotemporal dynamics through four change codes: persistent unsuitable (0–0), suitability gain (0–1), suitability loss (1–0), and persistent suitable (1–1). We tracked the center of mass of sect. *Camellia* to measure habitat migration, using the SDM toolbox (the GIS toolkit operating based on Python) to simulate and compare the geometric center shifts of suitable areas, revealing the effects of environmental changes on its distribution (Kong et al. 2021).

## 3 Results and Analysis

### 3.1 Model Optimization Results and Accuracy Evaluation

**FIGURE 2:**
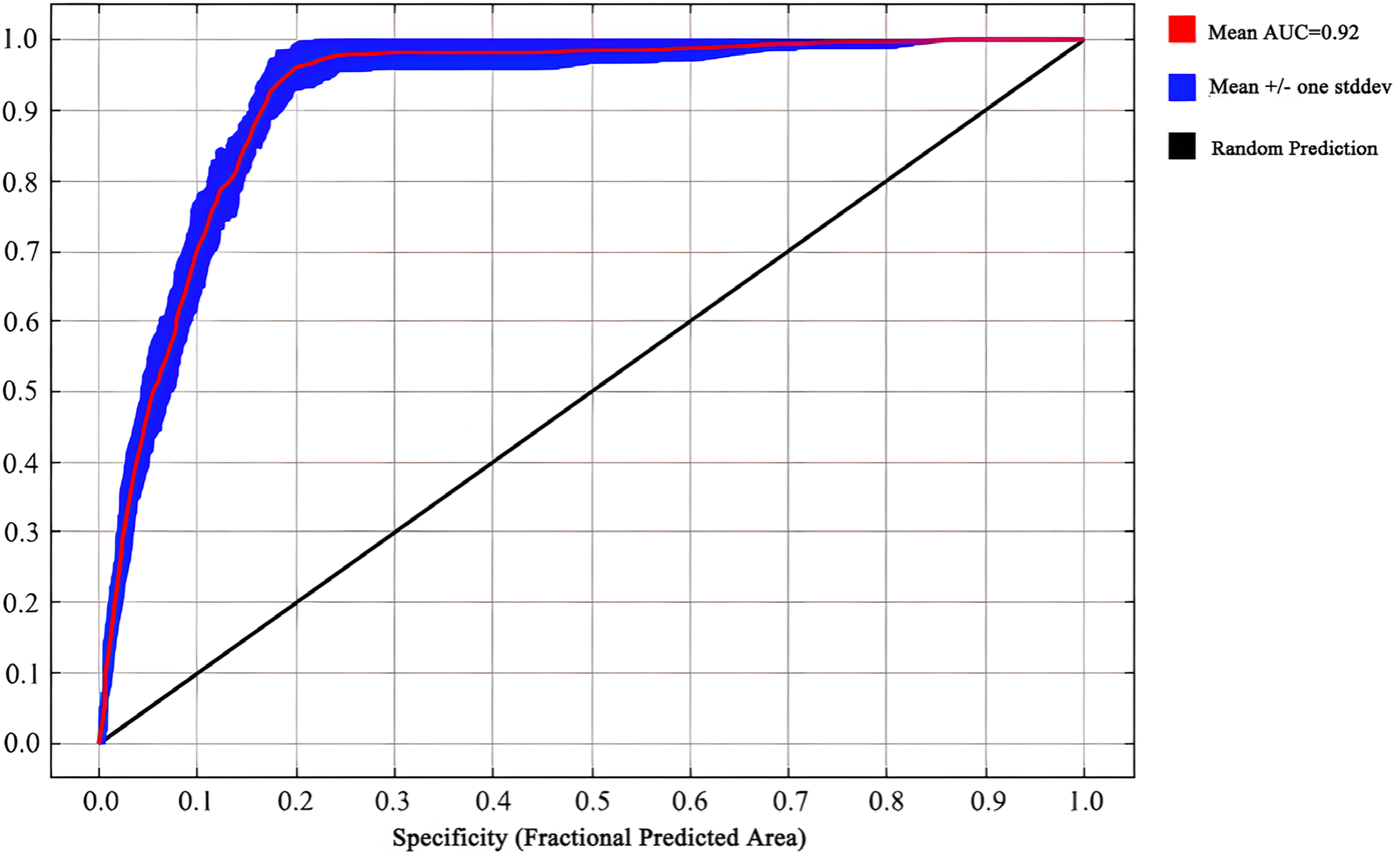
ROC curve of MaxEnt model prediction for sect. *Camellia* validation.

An RM value of 2.5 results in the FC being equal to LQHPT, which significantly reduces the complexity of the model and prevents overfitting, which in turn improves the accuracy of the model. The accuracy of this optimized model was evaluated by means of ROC curves. The average AUC values of the model were maintained within a stable interval of 0.920 ± 0.15 under various past, present, and future climatic conditions (Figure 2). This experimental result confirmed that the adjusted MaxEnt model exhibits smooth response curves consistent with the model's setup, and its AUC value is ≥ 0.9, indicating excellent predictive performance. The Kappa and TSS values exceeded 0.7 (Table 3).

**TABLE 3:** The values of AUC, Kappa, and TSS.

Category	Value
AUC	0.920
Kappa	0.852
TSS	0.847

### 3.2 Main Climatic Factors Affecting the Geographical Distribution of Sect. *Camellia*

**FIGURE 3:**
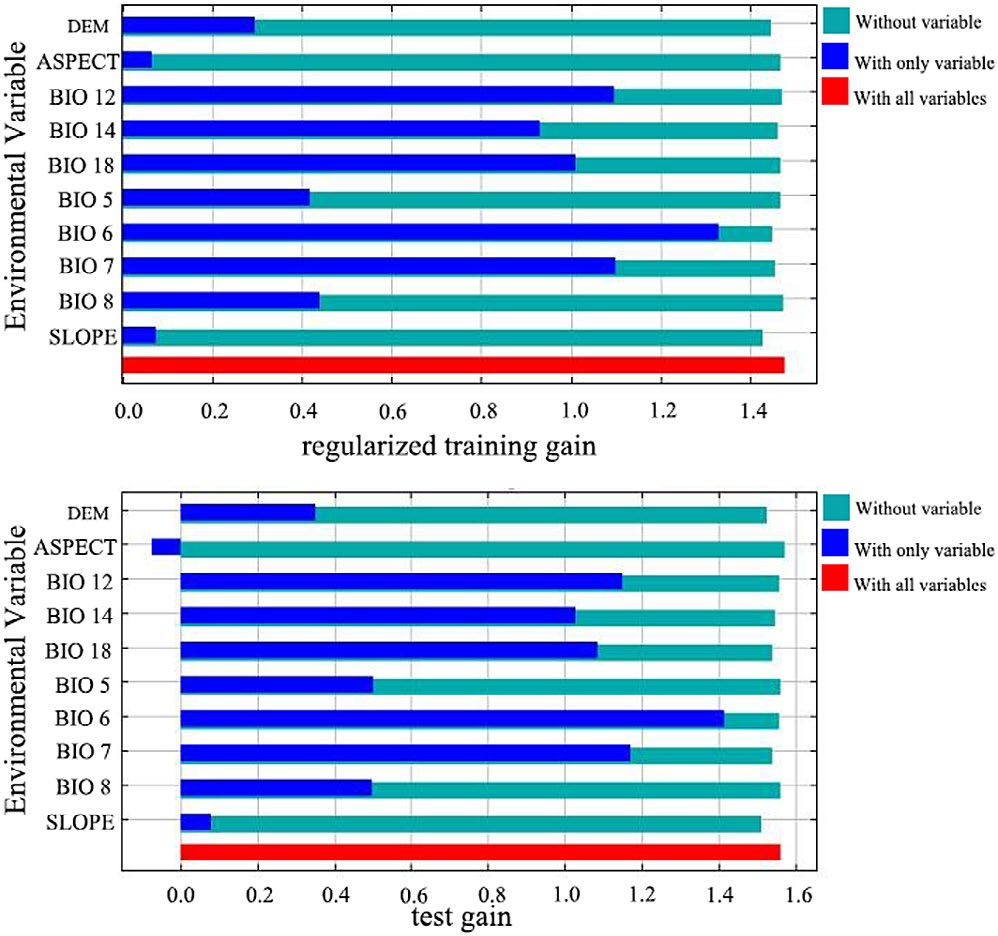
Jackknife method for evaluating major environmental factors.

**FIGURE 4:**
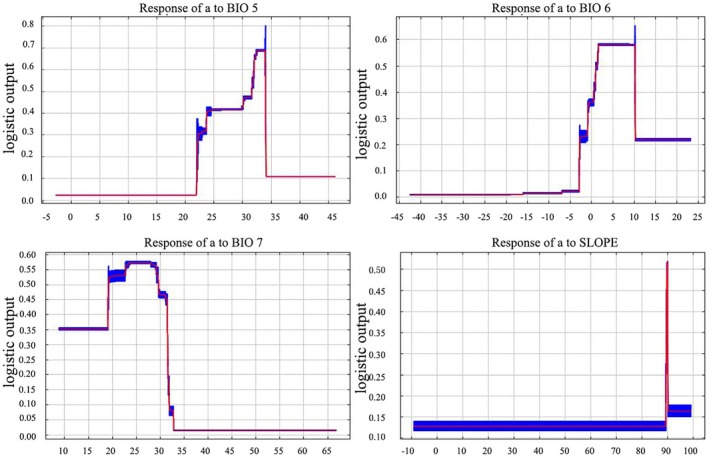
Relationships between potentially suitable areas and single‐factor response variables.

We analyzed the alternative contribution of variables and created response curves for individual factors. The data in Table 4 and Figures 3 and 4 indicate that the maximum temperature of the hottest month (Bio5) contributes 1.1% of the influence to the model, the interannual variation in temperature (Bio7) contributes 13.6%, and the slope of the terrain (Slope) contributes 3.3%. However, the minimum temperature of the coldest month (Bio6) was the most influential factor, contributing 78.2%, suggesting that it is a key environmental variable in determining the distribution of plants in sect. *Camellia*.

**TABLE 4:** Importance of each dominant environmental variable in the MaxEnt model.

Environmental variables	Description	Contribution (%)	Suitable range	Peak value
Bio6	Min temperature of coldest month	78.2	≥ −2.82°C	6.76°C
Bio7	Temperature annual range	13.6	≤ 31.61%	27.97°C
Slope	Slope	3.3	89.39°–90.04°	89.93°
Bio5	Max temperature of warmest month	1.1	22.04°C–34.17°C	33.92°C

We conducted separate simulation analyses for each of these four key environmental variables. By plotting response curves for a single factor, we were able to depict how the predicted probability of plant presence in sect. *Camellia* varied with each environmental factor. These response curves can be used to visualize the dynamic association between the distribution probability of a species' suitable survival area and the major environmental factors, thus providing insight into the ecological adaptations of these plants. The single‐factor response curves clearly reflect the relationship between the factors and the suitable areas; at the same time, we obtained the range of optimal values of the environmental variables from the tests, which clearly indicate the correlation between the existence probabilities and the environmental variables. The one‐way response curve reveals that when the maximum temperature of the warmest month (Bio5) is less than 22.5°C, the probability of the presence of sect. *Camellia* is 0; the probability of presence increases with increasing Bio5, and the probability of the distribution of sect. *Camellia* reaches a peak when Bio5 equals 33.92°C, which is the optimum suitable temperature (Figure 4). Taking a probability greater than 0.5 as the optimum range, the optimum range for the lowest temperature of the coldest month (Bio6) was greater than −2.82°C, and the highest peak value was 6.76°C. For the annual temperature range (Bio7), similar to that of Bio6, the probability of the existence of sect. *Camellia* increased with increasing temperature, with an optimum peak of 27.97°C and a suitable growth range of 19.5°C–30°C. When Bio7 is equal to 32.5°C, the distribution probability of sect. *Camellia* is infinitely close to 0. For the slope (Slope), the suitable range is 89.39°–90.04°, and the highest peak value is 89.93°.

### 3.3 Potential Habitable Areas of Sect. *Camellia* in Contemporary Climates

**FIGURE 5:**
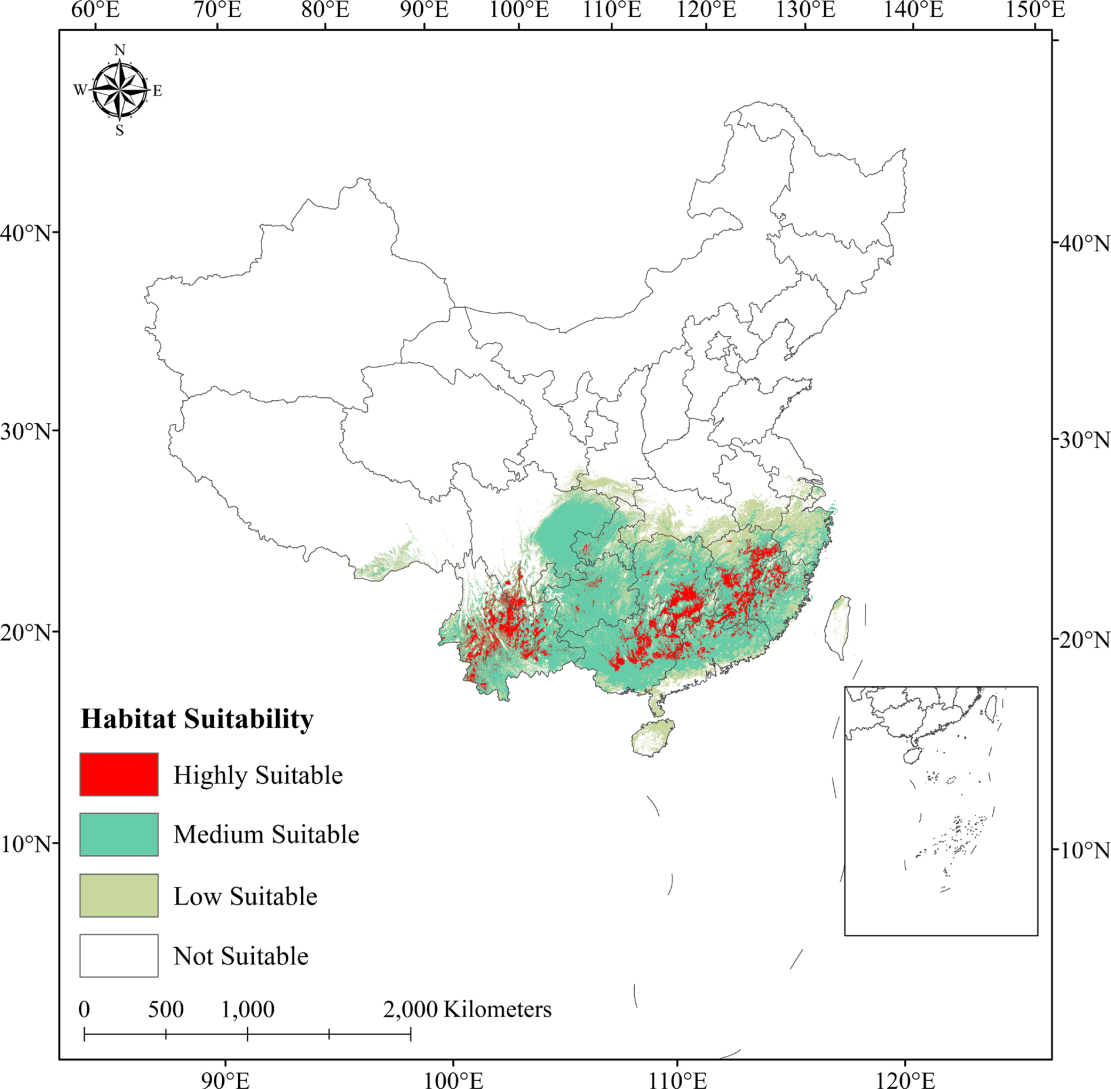
Potential habitat areas of sect. *Camellia* in China under current climatic conditions.

The main distribution range of the plants in sect. *Camellia* is 21.57°–31.48° N, 97.85°–127.54° E. They are concentrated in Yunnan, Guizhou, and Sichuan in Southwest China, Hunan in Central China, Guangdong in South China, Zhejiang in East China, and coastal areas. Under contemporary climatic conditions, the total habitable zone, that is, the main distribution area of sect. *Camellia*, is concentrated in parts of southwestern China and southeastern China, with a total habitable zone area of 17.04 × 10^5^ km^2^ (Figure 5).

(1) *Highly suitable zone*: This area is mainly distributed throughout the entire territory of Yunnan, central and eastern Guangxi, southern Hunan, Guangdong, Jiangxi, and western Zhejiang, with a total area of 1.95 × 10^5^ km^2^, accounting for 11.44% of the total adapted area; this area is also present on the west side of China, in Yunnan, southeastern Nanjing, the middle and lower reaches of the Yangtze River, and the Mount Wuyi. (2) *Moderately suitable zone*: This zone is mostly located around the highly suitable zone and is mainly distributed in eastern Yunnan, Guizhou, eastern Sichuan, Chongqing, Guangxi, Guangdong, Hunan, Fujian, and Jiangxi junctions and Zhejiang areas in China. The moderately suitable zone covers an area of 9.83 × 105 km^2^, accounting for 57.69% of the total suitable area. (3) *Low suitability zone*: This zone is mainly distributed at the junction of Gansu and Sichuan and in the southern parts of Hubei, Anhui, Jiangsu, and Hainan. The area of the low suitability zone is 5.26 × 10^5^ km^2^, accounting for 30.87% of the total area of the suitable zone.

### 3.4 Simulation of Past and Future Potentially Suitable Areas for Sect. *Camellia*

**FIGURE 6:**
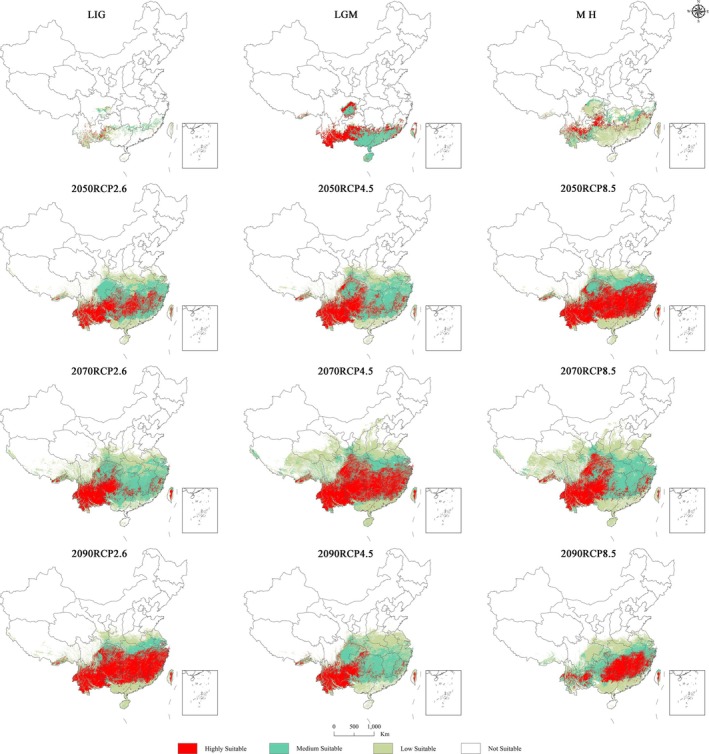
Changes in the spatial patterns of suitable areas for sect. *Camellia* under different climate scenarios.

According to the results (Table 5, Figure 6), from the LIG period to the contemporary period, the total habitable area of sect. *Camellia* showed a continuous increasing trend, expanding from 2.68 × 10^5^ km^2^ (LIG) to 7.68 × 10^5^ km^2^ (LGM) and then to 10.47 × 10^5^ km^2^ (MH). In this process, the potential total habitable zone area peaked, while the low and medium suitability zones showed similar trends, and the highly habitable zone gradually increased.

**TABLE 5:** Suitable areas for sect. *Camellia* in different time periods.

Climate scenarios	High (×10^5^ km^2^)	Medium (×10^5^ km^2^)	Low (×10^5^ km^2^)
Last interglacial	0.26	0.68	1.74
Last glacial maximum	3.12	3.78	0.78
Mid‐Holocene	1.52	2.37	6.58
Current	1.95	9.83	5.26
2050‐RCP2.6	6.11	8.67	6.57
2050‐RCP4.5	6.44	9.55	6.19
2050‐RCP8.5	11.91	5.91	5.63
2070‐RCP2.6	5.75	10.55	8.74
2070‐RCP4.5	13.03	8.36	13.68
2070‐RCP8.5	7.83	11.77	14.86
2090‐RCP2.6	12.3	5.33	6.68
2090‐RCP4.5	4.7	9.56	8.67
2090‐RCP8.5	5.83	6.35	8.23

The total area suitable for growing sect. *Camellia* shows a gradual expansion over time, that is, from the current period to the future year 2100. Under one of the three different climate change scenarios (RCP2.6, RCP4.5, RCP8.5), It is projected that by the 2050s, 2070s, and 2090s, the cumulative area suitable for growth is projected to exceed 2.1, 2.5, and 2.0 million square kilometers, respectively (Table 4, Figure 5).

Specifically, under the scenario setting of 2050s RCP 2.6, the total suitable growing area under the different climate scenarios is projected to exceed 21.35 × 10^5^ km^2^, of which the highly suitable growth area is about 6.11 × 10^5^ km^2^, which are increases of approximately 4.31 × 10^5^ and 4.06 × 10^5^ km^2^, respectively, compared with the current situation, whereas under RCP4.5, the total suitable growing area is projected to expand to 22.18 × 10^5^ km^2^, an increase of approximately 5.14 × 10^5^ km^2^ compared with the current scenario, whereas the area of the highly suitable growing area is projected to increase from the current 1.95 × 10^5^ to 2.28 × 10^5^ km^2^. Finally, under the most extreme RCP8.5 scenario, the total suitable growing area is projected to reach 23.45 × 10^5^ km^2^, and the highly suitable growing area is projected to increase to 11.91 × 10^5^ km^2^, which are increases of 6.41 × 10^5^ and 9.96 × 10^5^ km^2^, respectively, compared with the current scenario.

In the 2070s, the total suitable growing area under the different climate scenarios is projected to exceed 2.5 million km^2^. For example, under the RCP2.6 scenario, the total suitable growing area is expected to reach 25.04 × 10^5^ km^2^, and the highly suitable growing area is expected to reach 5.75 × 10^5^ km^2^, which are 8 × 10^5^ and 3.8 × 10^5^ km^2^ greater than the contemporary areas, respectively. In the RCP4.5 scenario, the total suitable growing area reaches 35.07 × 10^5^ km^2^, which is almost twice as large as the contemporary area; the highly suitable growing area is 13.03 × 10^5^ km^2^ and the low suitability area is 18.03 × 10^5^ km^2^, which are 11.08 × 10^5^ and 5.88 × 10^5^ km^2^ larger than the contemporary area.

By the 2090s, the total suitable growing area under all three climate scenarios exceeded 2 million km^2^. Under the RCP2.6 scenario, for example, the total suitable growing area reaches 24.31 × 10^5^ km^2^, an increase of 7.27 × 10^5^ km^2^ compared with that in the contemporary period, and the highly suitable growing area is 12.3 × 10^5^ km^2^, which is also an increase of 7.27 × 10^5^ km^2^. Under the RCP4.5 scenario, the total suitable growing area covers 22.93 × 10^5^ km^2^, an increase of 5.89 × 10^5^ km^2^ compared with the contemporary period. The highly suitable growing area is 4.7 × 10^5^ km^2^, which is an increase of 2.75 × 10^5^ km^2^. For the RCP8.5 scenario, the total suitable growing area is 20.41 × 10^5^ km^2^, which is an increase of 3.37 × 10^5^ km^2^ compared with that in the contemporary period, and the highly suitable growing area is 5.83 × 10^5^ km^2^, which is an increase of 3.88 × 10^5^ km^2^.

In summary, the potentially highly suitable distribution areas in the nine different future scenarios all have increasing trends when compared with those in the contemporary period. In the 2050s, the highly suitable area increased in southern China, bounded by Yunnan toward Guizhou, Hunan, Zhejiang, and Jiangsu. Figure 6 shows that there is some variability in the optimal growing regions of sect. *Camellia* over time. Nevertheless, the core distribution zone is still concentrated in southwestern China, especially in Yunnan Province, Guizhou Province, Guangxi Zhuang Autonomous Region, Hunan Province, Jiangxi Province, and Hubei Province, which constitute the main suitable growing areas for sect. *Camellia*.

### 3.5 Changes in the Spatial Pattern of Potential Habitats for Sect. *Camellia*

**FIGURE 7:**
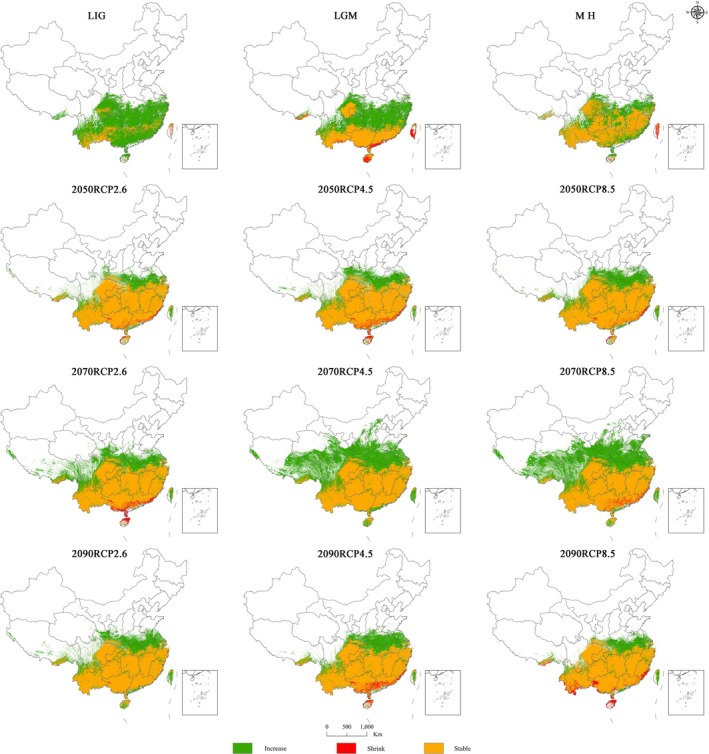
Trends of increases and decreases in the plant suitability zones for sect. *Camellia* under different climate change scenarios.

According to the data in Table 6 and Figure 7, the spatial pattern of the habitable zone for sect. *Camellia* showed an increasing trend over the past three periods when compared with the contemporary period. The LIG period had the largest added area of 15.69 × 10^5^ km^2^, while the smallest reserved area was only 2.91 × 10^5^ km^2^, and the lost area was 0.08 × 10^5^ km^2^. The rates of increase and loss of suitable zones were 92.07% and 4.69%, respectively. During this period, the increases in area were distributed mainly in central Yunnan, Guizhou (except for the southern portion), Guangxi, Guangdong, Chongqing, eastern Sichuan, Hunan, Jiangxi, Fujian, Zhejiang, Shanghai, and northeastern Hainan, whereas the loss of areas was mainly concentrated in some areas of Taiwan and southern Hainan. The area gained during the last glacial bloom was 10.79 × 10^5^ km^2^, the area lost was 0.75 × 10^5^ km^2^, and the rates of increase and loss of suitable area were 63.32% and 4.40%, respectively. This finding indicates that under the climatic conditions of the LIG period, the suitable area for sect. *Camellia* is quite different from that in the contemporary period, and a stable zone of sect. *Camellia* has formed in southern China. The prediction model reveals that the new area is mainly distributed in Guangxi, north‐central Guizhou, the part of Sichuan bordering Chongqing, Hunan, southern Hubei, Jiangxi, Zhejiang, Shanghai, and other areas. Owing to the increase in global temperatures, southern China may become a suitable area for sect. *Camellia*, while the loss of areas is concentrated mainly in the southern Guangdong, southern Guangxi, Hainan, and Taiwan sections. By the MH, the minimum area gain was 7.36 × 10^5^ km^2^, the loss area was 0.3 × 10^5^ km^2^, and the maximum area of the reserved area was 11.26 × 10^5^ km^2^. The main loss area was concentrated in Taiwan. Compared with the previous period, the model predicted fewer regional differences in the new areas, with rates of increase and loss of suitable areas of 43.19% and 1.76%, respectively.

**TABLE 6:** Changes in the size of the suitable area for sect. *Camellia* during each time period.

Climate scenarios	Increase (×10^5^ km^2^)	Stable (×10^5^ km^2^)	Shrink (×10^5^ km^2^)
Last interglacial	15.69	2.91	0.08
Last glacial maximum	10.79	7.83	0.75
Mid Holocene	7.36	11.26	0.3
2050‐RCP2.6	5.02	18.23	0.47
2050‐RCP4.5	6.23	17.82	0.87
2050‐RCP8.5	7.13	18.29	0.4
2070‐RCP2.6	9.25	17.81	0.88
2070‐RCP4.5	18.75	18.67	0.03
2070‐RCP8.5	18.56	18.16	0.53
2090‐RCP2.6	7.76	18.6	0.09
2090‐RCP4.5	7.12	17.69	1
2090‐RCP8.5	4.71	17.49	1.2

Compared with the contemporary period, the reserved area does not differ much under the 12 different future climate scenarios, all of which remain at 17.49 × 10^5^–18.67 × 10^5^ km^2^. The largest increase in area is 18.75 × 10^5^ km^2^ in the 2070s under the RCP4.5 scenario, with the additional areas mainly distributed in Yunnan, southern and eastern Sichuan, Chongqing, Hubei, southern Shaanxi, southwestern Henan, Anhui, Jiangsu, and Taiwan. The area with the least area added is 4.71 × 10^5^ km^2^ in the 2090s under the RCP8.5 scenario, with the areas concentrated in Hubei, Anhui, and southern Jiangsu. The loss area is maintained at 0.03 × 10^5^–1.2 × 10^5^ km^2^, with the largest loss occurring in the 2090s under the RCP8.5 scenario at 1.2 × 10^5^ km^2^, and the areas of loss are located in the very southern part of Yunnan, a few western parts of Guizhou, and Hainan. In the 2070s under the RCP4.5 scenario and in the 2090s under the RCP2.6 scenario, there are the smallest loss areas of only 0.03 × 10^5^ and 0.09 × 10^5^ km^2^, respectively.

Overall, under future climate scenarios, the concentration of potential areas suitable for distribution of sect. *Camellia* increases, the degree of fragmentation decreases, and the loss of its distribution area is concentrated mainly in southern Guangxi, southern Guangdong, and Hainan. The increase in the area is mainly toward the border areas of Hunan, Hubei, and Jiangxi Provinces, indicating that these areas may be sensitive to changes in future patterns and should be emphasized.

### 3.6 Direction of Center‐of‐Mass Migration in the Habitable Zone

**FIGURE 8:**
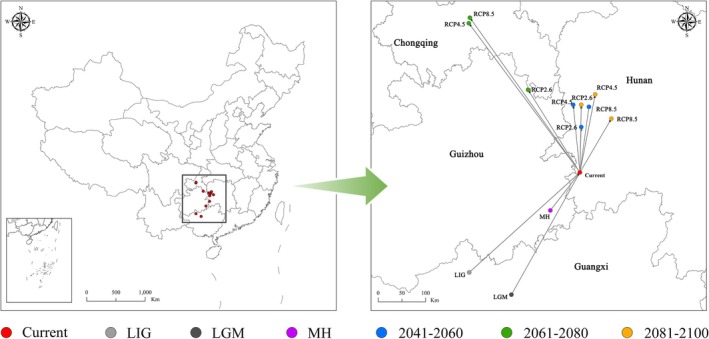
Center of mass offset positions of the aptitude zones of sect. *Camellia* under different climatic scenarios at different times.

By defining the potential fitness distribution area of sect. *Camellia* in terms of geometric centroids, it is possible to simulate the migratory changes in the center of mass under different climatic scenarios (Table 7 and Figure 8). According to the data and graphs involved, the center of mass of the suitable area for contemporary plants of sect. *Camellia* is located in the western part of Huitong County, Hunan Province, bordering Tianzhu County, Guizhou Province. In contrast, during the last three historical periods (LIG, LGM, and MH), the center of mass was located in southwestern China relative to the contemporary position: southeastern Guizhou and northwestern Guangxi. During the LIG period, the center of mass moved a distance of 0.31 × 10^3^ km and was located near Tian'e County, Guangxi, which is the farthest distance from the contemporary position and indirectly shows that climate change is obvious. In contrast, the migration distances during the LGM and the MH were 0.29 × 10^3^ and 0.1 × 10^3^ km, respectively, and were located near present‐day Baitu Township, Hechi City, Guangxi, and near Liping County, Guizhou Province, respectively. Overall, over time, the past three historical periods revealed a northward migration trend that continued into the contemporary era.

**TABLE 7:** Center of mass offset distances of sect. *Camellia* at different periods of time.

Phase		Longitude/(°)	Latitude/(°)	Distance/(×10^3^ km)
Last interglacial		107.24	24.99	0.31
Last glacial maximum		108.08	24.55	0.29
Mid‐Holocene		108.95	26.09	0.1
Current		109.61	26.76	0.00
2050s	RCP2.6	109.69	27.61	0.1
	RCP4.5	109.55	28.04	0.15
	RCP8.5	109.88	27.98	0.14
2070s	RCP2.6	108.61	28.36	0.21
	RCP4.5	107.38	29.67	0.39
	RCP8.5	107.41	29.77	0.4
2090s	RCP2.6	109.72	28.03	0.14
	RCP4.5	110.03	28.21	0.17
	RCP8.5	110.34	27.73	0.13

According to this study, by 2050, depending on different scenarios, the center of mass will migrate northward relative to the contemporary position, with a particular focus on the western part of Hunan. In the 2050s RCP2.6 scenario, the center of mass is closest to the contemporary position, 0.1 × 10^3^ km in Zhijiang Dong Autonomous County; in the 2050s RCP4.5 scenario, the center of mass is located in Mayang Miao Autonomous County; and in the 2050s RCP8.5 scenario, the center of mass is located in the southwestern section of Chenxi County. By 2070, the center of mass will shift northeastward relative to that in 2050, with the center of mass located in Yanjiang Tujia Autonomous County in northeastern Guizhou bordering Chongqing under the RCP2.6 scenario, whereas the center of mass will shift to the Fuling County area in south‐central Chongqing under the RCP4.5 and RCP8.5 scenarios, with 2070s RCP8.5 being farthest from the contemporary location at 0.4 × 10^3^ km. By 2090, the overall center of mass will shift southeastward on the basis of the 2070 position and will be in Chenxi County as well as Xupu County in eastern Huaihua, Hunan.

In general, under future climate scenarios, the center of mass will move northward, and the suitable areas will be mainly concentrated in the middle and lower reaches of the Yangtze River, southeastern hills, and Wuling Mountains in western Hunan, which suggests that the future distribution of sect. *Camellia* will shift to higher latitudes under the influence of future climate scenarios.

## 4 Discussion

### 4.1 Dominant Environmental Factors Affecting the Distribution of Sect. *Camellia*

In the field of ecological niche modeling, the MaxEnt model is widely used for species distribution prediction, especially in studies of ecology, conservation biology, and evolutionary ecology, and is favored for its ability to produce stable and reliable predictions on small sample data (Li, Shao, et al. 2022). Popular in species distribution studies based on occurrence records and randomly generated background points, the model can generate robust and accurate predictions even when small sample sizes are used (Xu et al. 2023). Nevertheless, the MaxEnt model focuses more on the basic ecological needs of a species than its actual dispersal ability; thus, the resulting potential distribution range may be broader than the actual distribution range (Cui et al. 2023; Wang et al. 2022). To optimize the model parameters, the Kuenm data package was used in this study, and the excellent performance of the model was verified by the AUC values, which revealed that the predicted suitable habitats of sect. *Camellia* plants were highly consistent with their actual distributions. By analyzing four key environmental factors—minimum temperature in the coldest month (Bio6), annual temperature difference (Bio7), maximum temperature in the warmest month (Bio5), and slope—we found that temperature was the most influential factor, which is in line with previous studies on the climate adaptation of sect. *Camellia* (Mo 2015; Miao et al. 2024). Sect. *Camellia* grows mainly in southern China, especially in Hunan, where it spreads to neighboring provinces, and its distribution in the Yunnan–Guizhou Plateau section reflects the subtropical monsoon climate of the region (Su 2021). In eastern China, on the other hand, sect. *Camellia* is found in the transition zone from the Jiangnan hills to the Jianghan Plain, where climatic conditions are particularly suitable for plant growth, especially the temperature factor, which is crucial in determining the distributional boundaries of tropical and subtropical species (Zhong et al. 2023).

### 4.2 Changes in Spatial Distribution Patterns and Center‐of‐Mass Analyses of Potential Habitat Areas of Plants in Sect. *Camellia*

The AUC value of the MaxEnt model test achieved 0.920, signifying the model's predictions are highly accurate and reliable. The model's predictions indicated that the optimal distribution area for contemporary sect. *Camellia* is primarily concentrated in most regions of East China, characterized by a humid subtropical monsoon climate, aligning with the actual natural distribution points of sect. *Camellia*. An analysis of the potential distribution patterns of sect. *Camellia* under climate change scenarios revealed its significant role in species conservation and ecological balance maintenance. Different species exhibit distinct distribution patterns in response to climate change. Under future climate scenarios, the suitability zone for sect. *Camellia* expanded significantly from South China to North China, focusing in Central, East, and South China and encompassing nearly the entire territories of Zhejiang, Jiangxi, Guizhou, Hunan, and Guangdong. This expansion underscores the high precision and optimal performance of the optimization‐based MaxEnt model in predicting sect. *Camellia*'s distribution, consistent with previous studies on the geographic distribution of sect. *Camellia* (An 2006; Feng et al. 2016; Yang et al. 2022). Climatic conditions dictate species distribution, and the distribution characteristics of species, in turn, reflect different climatic features (Li, Zhao, et al. 2022). From the LIG period to the contemporary period, the habitable zone area for sect. *Camellia* has shown a consistent increasing trend, reaching its maximum in the contemporary period, suggesting some expansion during this time. This expansion is hypothesized to be related to increased CO_2_ emissions and intensified human activities, such as logging. In the next three periods, the potential habitable zone for sect. *Camellia* is expected to increase to varying degrees under different climate scenarios compared to the contemporary period. It is speculated that sect. *Camellia* may be better adapted to future climatic conditions with gradual temperature increases. However, in future distribution patterns, low‐concentration emission scenarios in 2070 and 2090 are expected to have a relatively low rate of habitat loss, while high‐concentration emission scenarios are anticipated to have a relatively high rate of loss. This is speculated to be possible under future climate conditions, indicating that environmental degradation caused by high‐concentration emission scenarios may exceed the growth range of sect. *Camellia* field populations, impacting their survival probabilities and leading to an increase in the area of potential habitable areas compared to the decrease observed in low‐ and medium‐concentration emission scenarios. Therefore, this study also suggests that, in the context of global warming, the niche area of sect. *Camellia* will expand without anthropogenic disturbances and will increase with rising temperatures, potentially leading to a decline or extinction of the plant.

The center of mass migration analysis reveals that the contemporary habitable zone for sect. *Camellia* is centered in western Huitong County, Hunan Province. Historically, this zone has shown a northward shift, with a contemporary trend toward the north and northwest. In line with global warming, the potential distribution area's center of mass for sect. *Camellia* is moving toward higher latitudes, supporting the findings on plant migration in response to warming climates (An et al. 2023). Sect. *Camellia* has adapted to high mountain, Karst, and Yangtze River Basin environments, with climate warming potentially leading to wetter conditions at higher latitudes and drier climates at mid‐latitudes. This aligns with observations on the expansion of suitable habitats to higher altitudes in southern China (Du et al. 2021). The migration of sect. *Camellia* populations to higher latitudes is a key survival strategy against climate change, with potential for future spread to lower altitudes. Comprehensive future studies should integrate physiological, biochemical, and anthropogenic factors for a holistic assessment of sect. *Camellia*'s geographic distribution. The study concludes that while the total suitable area for sect. *Camellia* fluctuates, but highly suitable areas remain stable, indicating high adaptability to climate change and a substantial, enduring natural habitat, akin to findings on *Pinus sylvestris* distribution (Duan et al. 2022).

### 4.3 Limitations of the Study

#### 4.3.1 Data Sources

GBIF serves as a portal for global biodiversity information, and its data collection and storage may vary across regions, which can lead to sampling bias in species occurrence records (Beck et al. 2014). For example, some regions may have more intensive sampling sites due to the abundance of species resources, while other regions may have sparse data due to limited resources. This uneven sampling may affect the accuracy and reliability of model predictions. Second, while the data provided by WorldClim cover a wide range of climatic variables, they are based on observations from meteorological monitoring stations and generated through interpolation methods. Therefore, these data may not fully capture small‐scale environmental changes or microclimatic features under specific habitat conditions, and these errors may affect model results in model predictions (Poggio et al. 2018; Cerasoli et al. 2022).

Therefore, our study must carefully consider these potential data biases and limitations when utilizing GBIF and WorldClim data. Future studies can further optimize the predictive power of the models by adding ground validation data and improving the spatial resolution of the data. Also, the evaluation of the model should include a sensitivity analysis of the bias of the data sources to ensure the robustness of the study results.

#### 4.3.2 Limitations of the Model

The MaxEnt model assumes that species are in equilibrium with their surroundings, a core principle of its modeling. Yet, this assumption may not hold in rapidly changing climates or for species with poor dispersal abilities. In such scenarios, species may not have adjusted to current conditions, causing model predictions to be biased. Climate change can swiftly alter species distributions, outpacing migration rates and making equilibrium‐based predictions inaccurate. Similarly, species with limited dispersal might not reach suitable habitats, even if they appear environmentally suitable (Helmstetter et al. 2020).

Species distribution prediction models can lead to inaccurate predictions when a genus‐level modeling approach is used when dealing with species with specific environmental requirements. This is because such models usually assume a linear relationship between species and environmental variables and use the same environmental variables and parameter settings for all species in the prediction process. This approach ignores subtle differences in the ecological requirements of different species, such as specific preferences for temperature, precipitation, and soil type. Therefore, when predicting the distribution of species with differentiated ecological requirements, these models often have difficulty accurately reflecting the unique ecological niches of each species, limiting the effectiveness of their application in complex ecosystems (Claerhout et al. 2023).

Our study must consider these constraints when using MaxEnt and interpret results cautiously. Future studies can enhance the model's applicability and prediction accuracy by integrating more ecological and biological data and by regularly testing its assumptions.

#### 4.3.3 Impact of Uncertainty in Climate Projections

When examining how future climate scenarios might affect the sect. *Camellia* formation's habitats, we acknowledge the uncertainty in the climate models we use. The variations among general circulation models (GCMs) greatly influence habitat suitability predictions, requiring careful analysis (Davis et al. 2022). The diverse climate outcomes from GCMs and their varying responses to identical scenarios can result in inconsistent habitat projection results. Thus, it is crucial to account for GCM uncertainties when interpreting our findings and to employ a multi‐model approach to mitigate the bias from relying on a single model. This strategy allows us to more accurately evaluate the impacts of climate change on sect. *Camellia* habitats and to establish a stronger scientific foundation for conservation efforts.

#### 4.3.4 Generalization of Results

Our model faces a significant limitation due to the absence of experimental or field validation for its predictions. To enhance the model's predictive power, we suggest that future research should focus on expanding field surveys. This will help to fine‐tune the model, bolster its empirical foundation, and increase its academic credibility. By correlating field data with model outcomes and conducting cross‐validation across various settings, we can significantly improve the model's applicability and accuracy.

#### 4.3.5 Geographical and Ecological Constraints

This study did not fully investigate the geographical and ecological constraints of our model, which is crucial for understanding its generalizability across different regions and its applicability to closely related species. Species distributions are shaped by a variety of factors, including climate, topography, and biotic interactions, and those with limited dispersal abilities may struggle to adapt swiftly to environmental shifts (Frey 2010). To advance our model's predictive capabilities, future research should focus on validating it across various locations and testing its accuracy for closely related species. This will involve gathering more comprehensive distribution data and refining the model to enhance its broad applicability and predictive accuracy.

#### 4.3.6 Lack of Historical Validation

This study employed paleoclimatological methods to initially validate past distribution predictions. However, due to the large number of species in the sect. *Camellia* and the scarcity of fossil pollen evidence, we have yet to establish a complete historical validation cycle. Future work will focus on three main areas: (1) collaborating with paleoecologists to obtain additional borehole pollen data; (2) integrating ancient DNA metabarcoding techniques to reconstruct historical communities; and (3) developing a Bayesian validation framework that incorporates archaeological evidence (Flantua et al. 2023). These efforts aim to provide a more comprehensive understanding of the evolutionary history of *Camellia* sect. *Reticulatae* and inform future conservation strategies.

### 4.4 Practical Implications of the Study

This research provides operational frameworks for biodiversity conservation through predictive habitat modeling of sect. *Camellia*, enabling precise identification of climate‐vulnerable zones critical for sustainable land management. The model's spatial outputs facilitate evidence‐based decision‐making by quantifying anthropogenic climate impacts on species distribution, particularly relevant given projected range contractions under high‐emission scenarios. Three implementation pathways are proposed: (1) Establishment of multi‐stakeholder platforms for real‐time data sharing in high‐suitability regions, (2) development of adaptive conservation protocols with phased objectives and accountability matrices, and (3) implementation of iterative monitoring systems using AUC‐validated metrics to optimize management interventions.

The predictive‐validation synergy elevates ecological forecasting accuracy, establishing climate‐informed land zoning protocols essential for adaptive conservation frameworks. These integrated methodologies decode species distribution mechanics through habitat suitability modeling, generating evidence‐based blueprints for maintaining ecological integrity across development gradients—particularly vital given identified climate‐driven habitat contractions.

The RCP8.5 scenario exhibits dual ecological impacts—stimulating short‐term species expansion while compromising long‐term viability. This paradox aligns with Chinese flora studies: *Schima superba* (Theaceae) distribution is projected to concentrate south of the Yangtze River Basin (Ding et al. 2024), while *Camellia sinensis* demonstrates climate‐driven range expansion (Zhang, Li, et al. 2019), both reaffirming temperature/precipitation as key climatic constraints consistent with Liu et al.'s (2019) plant distribution modeling framework.

## 5 Conclusion

This study employs Kuenm‐optimized MaxEnt modeling to delineate sect. *Camellia* habitat dynamics under multiple climate scenarios. Current optimal habitats (1.704 million km^2^) cluster in Yunnan's bioclimatic nexus, extending to Guangxi's central‐eastern regions, southern Hunan, Guangdong, Jiangxi, and western Zhejiang. Core distribution zones (0.195 million km^2^, 11.44% of total suitable areas) are concentrated in the southeastern Nanling Mountains, Jinsha River Basin, Yangtze mid‐reaches, and Wuyi Mountain periphery, governed by thermal extremes (warmest month maximum and coldest month minimum temperatures), annual temperature range, and topographic slope.

Given current fragmented distribution patterns and acute anthropogenic vulnerability, strategic interventions should prioritize: (1) anthropogenic decoupling through habitat corridor restoration; (2) climate‐resilient management protocols tailored to regional bioclimatic gradients; and (3) phased conservation integrating ecological‐economic valuation matrices. Implementing adaptive conservation frameworks, particularly through establishing bioclimatic corridors across southeastern China's mountainous ecotones, could enhance meta‐population connectivity while optimizing ecosystem service provision.

## Author Contributions

**Weihao Gu:** formal analysis (lead), methodology (lead), writing – original draft (lead). **Xu Xiao:** formal analysis (equal), validation (supporting). **Zhaohui Ran:** formal analysis (equal), validation (supporting). **Chao Yan:** visualization (supporting). **Dongzhen Jiang:** formal analysis (supporting). **Lei Zhou:** visualization (supporting). **Mingtai An:** validation (supporting). **Zhi Li:** conceptualization (equal), supervision (equal).

## Conflicts of Interest

The authors declare no conflicts of interest.

## Supporting information


Data S1.

Data S2.

## Data Availability

All necessary data have already been provided in the main text and [Link], [Link].
